# Lymphohematopoietic Malignancies in Uranium Miners: Kulich et al. Respond

**Published:** 2007-04

**Authors:** Michal Kulich, Vladimír Řeřicha, Robert Řeřicha, David L. Shore, Dale P. Sandler

**Affiliations:** Faculty of Mathematics and Physics, Charles University, Prague, Czech Republic, E-mail: kulich@karlin.mff.cuni.cz; Health Institute of Uranium Industry, Pøíbram, Czech Republic; Center of Epidemiological Studies, Pøíbram, Czech Republic; Westat, Inc., Durham, North Carolina; Epidemiology Branch, National Institute of Environmental Health, Sciences, National Institutes of Health, Department of Health and Human Services, Research Triangle Park, North Carolina

We read with interest the comments by Möhner regarding the analysis and interpretation of the case–cohort study of Czech uranium miners (Řeřicha et al. 2006). He noted that our results do not agree with two recent German studies that also investigated the link between leukemia and radiation exposure in uranium miners. Kreuzer et al. (2004) conducted a mortality study based on death certificates (although combined with autopsy records) and reported standardized mortality ratios. As noted in our article (Řeřicha et al. 2006), studies relying on vital statistics underestimate the incidence of cancers such as chronic lymphocytic leukemia (CLL), which are not rapidly fatal or systematically diagnosed. For example, compare our 84 leukemia cases to the 95 cases reported by Kreuzer et al. (2004) that were based on a total follow-up period that was more than 4 times longer. The incidence rates or age differences between Czech and German miners cannot be that different. Möhner et al. (2006) published a well-designed, matched case–control study of cancer incidence with a large number of cases. Their reported results from grouped analyses and excess relative risk models indicated some elevated risk for CLL, which does not conflict with our conclusions. The lack of statistical significance can be explained by the relatively poor power of grouped analyses compared with the non-grouped Cox model we applied. Another important point that can explain seemingly conflicting results of different studies is the high sensitivity of the results to measurement error in exposures. A study that uses less precise estimates of radiation exposures is less likely to identify an existing exposure effect. This affects leukemia analyses more than lung cancer analyses because the effect of radon on lung cancer is much stronger and more difficult to miss.

In his letter, Möhner mentioned several other issues that need clarification. First, is stratification by duration of employment problematic, given the strong association of this variable with exposure? In fact, as shown, for example, by Borgan et al. (2000) and Kulich and Lin (2004), stratification on variables correlated with exposure is always highly desirable because it can substantially increase the precision of the analysis at little cost. As long as a correct procedure for estimating parameters from stratified samples is used, the estimates are valid and their standard errors are reduced. Stratification by age is a similar case; in these data, age is also strongly related to exposure.

The reasoning for including miners who worked < 12 months was that they represent a natural comparison group with low exposures. Many occupational studies exclude workers with short employment periods [Kreuzer et al. (2006) included those with ≥ 6 months exposure]. Both approaches have pros and cons. Including miners with short working periods may increase power and is relevant when the primary interest is to compare incidences at high exposures with those at low exposures. In contrast, miners who left their jobs early may have done so because of health reasons, which could induce a healthy-worker effect. We decided to include all miners before the data were analyzed, and we presented the planned analysis in our article (Řeřicha et al. 2006). We did a separate analysis of miners who worked > 12 months underground and found generally stronger radiation effects on incidence. For example, for CLL the estimated relative risks comparing 110 working level months (WLM) to 3 WLM would be 3.13 [95% confidence interval (CI), 1.22–8.08; *p* = 0.02] based on 39 cases and 1,596 subcohort subjects. The CI was wide but the conclusion was not changed.

The odds ratios (ORs) in Möhner’s Table 1 would look less extreme if the last three groups were combined. The OR of 7.16 is based on a single case and the OR of 0.29 is based on three cases. Hence, the alleged heterogeneity does not look very convincing to us. Finally, the issue of latency period and late follow-up was addressed by separate analyses based on time since exposure. As we reported (Řeřicha et al. 2006), exposures acquired > 25 years ago had no noticeable effect on current incidence, whereas the most recent exposures (2–15 years ago) showed the strongest association.

In conclusion, we believe that our study (Řeřicha et al. 2006) offers the important advantage of having included incident cases and that the analysis was appropriate. The conclusions of the study do not depend on whether or not the analyses are restricted to miners with longer working periods.
